# Clinical and histological sequelae of surgical complications in horizontal guided bone regeneration: a systematic review and proposal for management

**DOI:** 10.1186/s40729-020-00274-y

**Published:** 2020-11-26

**Authors:** John Rong Hao Tay, Xiaotong Jacinta Lu, Wei Ming Clement Lai, Jia-Hui Fu

**Affiliations:** 1 Discipline of Periodontics, National University Centre for Oral Health Singapore, 9 Lower Kent Ridge Road, Singapore, 119085 Singapore; 2 Department of Restorative Dentistry, National Dental Centre Singapore, 5 Second Hospital Ave, Singapore, 168938 Singapore; 3 Statistics Unit, National University Centre for Oral Health Singapore, 9 Lower Kent Ridge Road, Singapore, 119085 Singapore

**Keywords:** Bone regeneration, Bone transplantation, Wound healing, Complications, Histology

## Abstract

It is not uncommon to encounter post-surgical complications after horizontal guided bone regeneration (GBR). The primary aim of this review was to evaluate the incidence and types of complications that occur after horizontal GBR and propose management strategies to deal with these clinical situations. A secondary aim was to conduct a histomorphometric review of the wound healing process at sites that experienced post-surgical complications after GBR. A keyword search of MEDLINE, EMBASE, and the Cochrane Central Register of Controlled Trials for studies published in English from January 2015 to January 2020 was conducted for the primary aim and 23 studies were selected. A second search addressing the secondary aim was conducted, and five studies were included. Site-level analysis showed that the weighted mean incidence proportion of minor wound dehiscence and minor infections occurring at the augmented site was 9.9% [95% CI 6.4, 13.9, *P* < 0.01] and 1.5% [95% CI 0.4, 3.1, *P* = 0.21) respectively. Patient-level analysis showed minor and major complications occurring at a weighted mean incidence proportion of 16.1% [95% CI 11.9, 20.8, *P* = 0.01] and 1.6% [95% CI 0.0, 4.7, *P* < 0.01] respectively, while neurosensory alterations at the donor site was 7.0% [95% CI 1.3, 15.5, *P* < 0.01]. Subgroup analysis also revealed that the use of block grafts increased the incidence proportion of minor post-surgical complications, whereas a staged GBR procedure increased the incidence proportion of both minor and major post-surgical complications. Although exposure of the barrier membrane is often associated with less bone regeneration and graft resorption, the type of membrane used (resorbable or non-resorbable) had no statistically significant influence on any post-surgical complication. Histologically, a layer of fibrous connective tissue instead of bone is commonly observed at the interface between the native bone at the recipient site and the regenerated bone in cases with membrane exposure after GBR procedure. Minor wound dehiscence was the highest incidence proportion of post-surgical complications. Methods ranging from daily application of antiseptics, use of systemic antimicrobials, regular reviews, and total removal of the non-integrated biomaterials are commonly prescribed to manage these post-surgical complications in attempt to minimise the loss of tissue at the surgical site.

**Supplementary Information:**

**Supplementary information** accompanies this paper at 10.1186/s40729-020-00274-y.

## Introduction

Given the high long-term survival and success rates, dental implants are regarded as the treatment of choice when replacing missing teeth. In order to achieve desirable treatment outcomes, the implants must be placed in prosthetically driven positions to facilitate the fabrication of aesthetically acceptable and functional reconstructions that are maintainable and stable over time. Yet, this ideal situation might not be readily achievable due to the changes in bone volume that occurs within the first 3 months of tooth loss. As such, guided bone regeneration (GBR) has been proposed to restore the lost bone volume to accommodate the dental implant prostheses. In this surgical technique, bone cells are given a protected environment to populate and mature into functional living bone by excluding epithelial cells and connective tissue through the use of barrier membranes and bone grafts [1, 2]. Although it is highly predictable [3–8] in terms of bone gain, it is a relatively technique sensitive procedure. Therefore, it is not uncommon to experience post-surgical complications after a GBR procedure. Two systematic reviews [9, 10] have assessed post-surgical complications in patients who underwent horizontal GBR, but none of these reviews were designed to specifically identify and analyse the different types of complications that occur after the procedure. This review thus seeks to evaluate the incidence and types of complications that occurred after horizontal GBR procedures were performed and propose appropriate management strategies to deal with these clinical situations. Furthermore, there is scarce data on the histomorphometric presentation of a regenerated site with less than ideal healing due to post-surgical complications. As such, the secondary aim for this paper is to review the wound healing process at a site with history of complications arising from a failed GBR procedure.

## Materials and methods

### Search strategy

#### To address the primary aim

An electronic search of MEDLINE (PubMed), EMBASE, and Cochrane Central Register of Controlled Trials (CENTRAL) databases was performed using the following search terms: (“dental implants”[MeSH Terms] OR “dental implantation, endosseous”[MeSH Terms] OR “implant*”[tw]); (“bone regeneration”[MeSH Terms] OR “bone substitutes”[MeSH Terms] OR “alveolar ridge augmentation”[MeSH Terms] OR “bone transplantation”[MeSH Terms] OR “guided bone regeneration”[tw] OR “GBR”[tw] OR “onlay graft”[tw]); (“postoperative complications”[MeSH Terms] OR “complication*”[tw]). Boolean operators (OR, AND) were used to combine the searches. Hand searching of the included journals was also conducted to ensure completeness of the search.

Studies that were included were as follows: (a) observational (case-control, prospective cohort studies, and case series) or interventional (randomised controlled clinical trials) studies published in the English language from January 2015 to January 2020, (b) horizontal ridge defects were present, (c) horizontal GBR (defined as the use of a space-maintaining bone graft with a barrier membrane) was carried out in healed ridges, (d) post-surgical complications were reported as a primary or secondary outcome, and (e) studies had to recruit more than 15 human subjects for horizontal GBR and were planned for implant placement.

Studies were excluded if (a) other forms of bone augmentation, besides horizontal GBR, were performed at the same surgical site (e.g. vertical GBR, sinus augmentation, ridge split); (b) around fresh extraction sites or immediate implants or implants with bone dehiscence due to peri-implantitis; (c) had concurrent periodontal plastic surgeries (e.g. free gingival grafts, frenectomies) performed; and (d) patients with conditions or are on medications that interfere with bone metabolism (e.g. osteoporosis), a history of head and neck radiotherapy or have severe metabolic disorders (e.g. uncontrolled diabetes mellitus).

The review for the primary aim was prepared according to the Preferred Reporting Items for Systematic Review and Meta-Analyses (PRISMA) checklist [11].

#### To address the secondary aim

An electronic search of the PubMed database of the US National Library of Medicine was conducted using the following search terms: (“wound dehiscence” OR “dehiscence” OR “flap dehiscence” OR “membrane exposure” OR “graft exposure” OR “graft failure” OR “infection”) AND (“ridge augmentation” OR “GBR” OR “guided bone regeneration”) AND (“wound healing” or “healing”). Hand searching of the included journals was also conducted to ensure completeness of the search. Studies that were selected (a) were published in English from January 1965 to January 2020; (b) performed GBR procedures; and (c) reported on histological and/or morphometric outcomes of sites in humans or animals where compromised healing such as infection, graft exposure, and/or wound dehiscence had occurred. Studies associated with alveolar ridge preservation or periodontal regeneration of alveolar bone defects around teeth were excluded.

### Data extraction and management

Data was extracted by two reviewers (JT and JL) who independently screened the titles, abstracts, and full texts of the included studies. Full-text examination was done for studies with insufficient information from the titles or abstracts to make a definitive decision, and any disagreement between the reviewers was resolved through discussion. To address the primary aim, the data from the studies eligible for selection was then extracted into standardised forms which included: the study design, number of subjects, number of surgical sites, mean age, smoking status, periodontal status, bone graft and barrier membrane used, number of implants placed, staged/simultaneous surgery, implant survival and success rate, follow-up period, horizontal bone gained after surgery, graft resorption rate, and number and rate of complications at the augmented and donor sites subclassified into its different types of complications. To address the secondary aim, information extracted from each study included the study model, defect type, healing period, device used, clinical presentation, and histological presentation of the wound.

### Risk of bias assessment

Two assessment tools were used to assess the risk of bias for the primary aim of the review. For observational studies and interventional studies, quality assessment was done using the Risk of Bias in Non-randomized studies—of Interventions (ROBINS-I) tool and Cochrane risk-of-bias tool 2.0 (ROB 2) respectively, as recommended by the Cochrane Handbook for Systematic Reviews of Interventions 6.0 [12, 13]. Risk of bias for observational studies was assessed for bias (1) due to confounding, (2) in selection of participants, (3) in classification of interventions, (4) due to deviations for intended interventions, (5) due to missing data, (6) in measurements of outcomes, and (7) in selection of the reported result. Bias was categorised as low, moderate, serious, or critical. Risk of bias for interventional studies were assessed for bias (1) arising from randomisation, (2) deviations from intended interventions, (3) missing outcome data, (4) measurement of outcome, and (5) selection of reported result. Bias was categorised as low, high, or some concerns. Assessment of risk of bias was done independently by two reviewers (JT and JL) and any disagreement was resolved through discussion.

### Data synthesis

Meta-analyses were conducted to estimate the overall incidence proportion with 95% confidence interval according to the different types of post-surgical complications, which include site-level analysis of minor wound dehiscences and minor infections at the augmented site, and patient-level analysis of the total minor and major complications occurring at the augmented site, and neurosensory alterations at the donor site. The incidence proportion was transformed using the Freeman-Tukey double arcsine transformation to stabilise the variance [14]. The random effects model was used in the meta-analyses to account for heterogeneity among studies. The statistical heterogeneity between studies was assessed using Cochran’s *Q* test and the *I*^2^ statistic. The *I*^2^ values of 25%, 50%, and 75% were considered to be of low, moderate, and high levels of heterogeneity respectively

The publication bias was evaluated with the funnel plot and the Begg-Mazumdar’s rank test [15]. To assess potential confounding factors, subgroup meta-analyses were performed to investigate the effect of the following variables: type of study, type of bone graft used (particulate or block), type of membrane used (resorbable or non-resorbable), and type of GBR technique (staged or simultaneous) on the different types of complications reported. The differences between subgroups were compared using the chi-squared test. All analyses were conducted using statistical software (R, version 3.63). A *P* value < 0.05 was considered statistically significant.

## Results

**Fig. 1:**
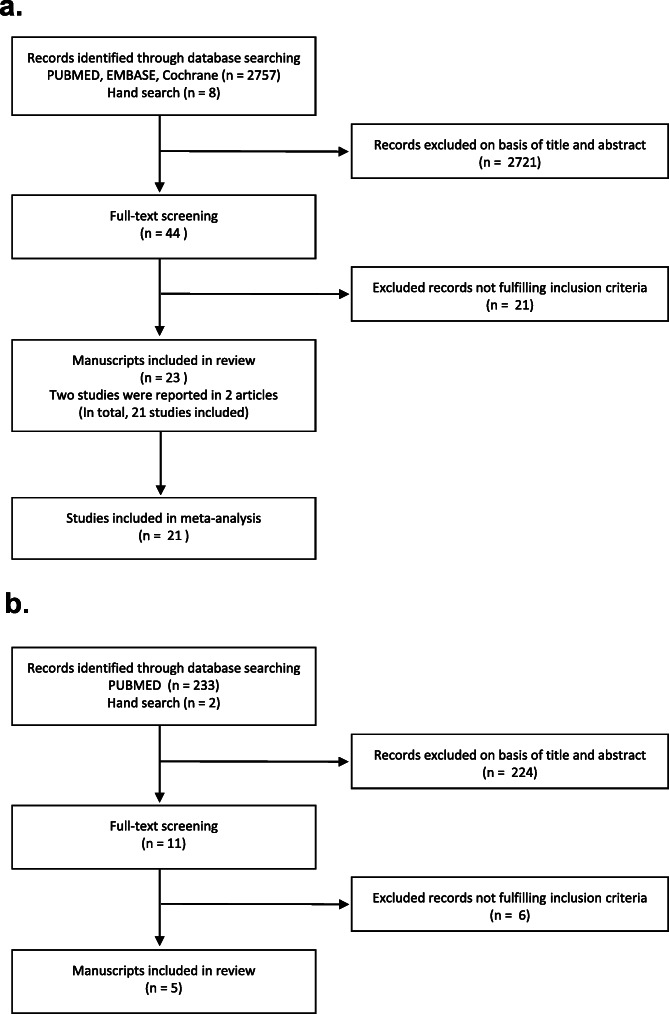
**a** Flow diagram of publications showing the study selection process for the primary aim of the study. **b** Flow diagram of publications showing the study selection process for the secondary aim of the study

A flow diagram depicting the selection process is shown in Fig. 1a. The database and hand search yielded a total of 2765 publications, of which 44 publications were selected for full-text examination, and 21 publications were excluded (Additional file 1). A final list of 23 publications fulfilled the selection criteria for the primary aim of this review. Meanwhile for the secondary aim, the database and hand searches yielded a total of 233 and two publications respectively. Thereafter, 11 publications were selected for full-text examination, but only five publications were eligible for final review (Fig. 1b).

### Study design and characteristics

Out of the 23 studies (Table 1), there were 11 randomised controlled clinical trials [21–24, 26, 29–32, 36, 37, 39], one prospective cohort study [34, 40], three retrospective cohort studies [18, 20, 33], and eight case series [16, 17, 19, 25, 27, 28, 35, 38, 41–43], of which two research groups independently published two follow-up papers on the same study cohort [21, 22, 27, 28], and they have been collectively grouped under one study name for this analysis. The follow-up period after ridge augmentation or implant placement was reported in all studies except two [16, 35], and it ranged from 3.1 months to 10 years. Two studies excluded smokers [30, 38], 14 studies reported periodontal status of subjects [16–18, 21–23, 25–30, 35, 36, 40–43], and excluded subjects with untreated periodontal disease, or with a full mouth plaque score and full mouth bleeding score of more than 25%.

**Table 1:** Features of included studies

Author/year	Study design	No. of subjects	Mean age ± S.D. (range)	Smoking status (%)	Periodontal status (%)	Test	Control	No. of implants placed	Staged/simultaneous GBR	Implant survival rate (%)	Implant success rate (%)	Follow-up period	Horizontal bone gained after surgery	Graft resorption	Baseline horizontal ridge crest thickness	Complications
Sakkas et al. 2016 [16]	Retrospective CS	112	34.3 (20–61.5)	74 smokers (66%) 38 non-smokers (34%)	7 generalised advanced periodontal disease, treated (6.3%) 105 periodontally healthy (93.7%)	Zygomatic ABG + rCM	NA	134	Staged	100%	100%	NR	NR	NR	NR	24 reports of incision line dehiscence, swelling, or wound infection (20 recipient sites, 4 donor sites) 2 pts: postop sinusitis with persistent fistula due to maxillary sinus perforation (donor site) 2 pts: (1.7%) temporary infraorbital nerve paraesthesia (donor site) 2 pts: (1.7%) total graft exposure with wound infection and suppuration Minor complications treated with CHX rinse and antibiotics (either orally or IV) Sinusitis treated surgically and with antibiotics Graft removed in 2 cases with total graft exposure Pts with paraesthesia were given follow-up until resolution at 6w
Wessing et al. 2016 [17]	Retrospective CS	36	57.7 ± 12.0 (32–76)	5 smokers (14%) 31 non-smokers (86%)	21 treated periodontal disease (58%) 15 periodontally healthy (42%)	pDBBM only or DBBM + pABG in 1:1 mixture vol + non-cross-linked CM	NA	103	7 (19%) staged, 29 (81%) simultaneous	100%	100%	18.3mo (6.3–28.6)	NR	NR	< 6 mm ridges were staged augmentations	6 dehiscences: 3 in simultaneous GBR cases, 3 in staged GBR cases 4 sites with spontaneous closure and no full membrane exposure or graft exposure 2 sites failed; required graft and membrane removals
Altiparmak et al. 2017 [18]	Retrospective cohort	48	44.8	NR	All periodontally healthy	Ramus block ABG + pDBBM + rCM	Ridge split + DBBM + rCM	42 (T) 43 (C)	Staged (T) Simultaneous (C)	92% (T) 100% (C)	NR	38.33 mo (T) 31.6 mo (C)	NR	NR	3–4 mm	T: 6 temporary graft exposures (14.3%); 3 mild infections (7.1%); 3 temporary paraesthesias (7.1%); 2 permanent graft exposures (4.8%) C: 1 temporary graft exposure (2.3%); 2 mild infections (4.7%); 3 bad split (7.1%)
Chappuis et al. 2017 [19]	Prospective CS	38	45 ± 13	5 light smokers (≤ 10 cig/day) (13%) 1 heavy smoker (3%) 32 non-smokers (84%)	NR	Symphysis/Ramus block ABG + pDBBM + rCM	NA	52	Staged	98.1%	98.1%	10y	3.16 ± 0.76 mm pre-op to 8.1 ± 1.02 mm post-op	− 0.34 mm (6.9%) (6mo); − 0.38 mm (7.7%) (10y)	≤ 5 mm	7 pts: temporary neurosensory disturbance at chin harvesting sites (3 at lower lip, 3 experienced negative pulp sensitivity of lower incisors, 1 had both), complete resolution between 2 and 6mo
Gurler et al. 2017 [20]	Retrospective cohort	50	31 (23–53)	NR	NR	Ramus block ABG + rCM	Ridge split + pDBBM + rCM	44 (T) 33 (C)	Staged (T, C)	93.1%	93.9%	3.3 (T) 3.1 (C)	1.8–2.7 mm (mean 2.5 mm) pre-op to 4.2–7.75 mm (mean 6.3 mm) post-op (T) 3.2–3.7 mm (mean 3.2 mm) pre-op to 4.0–7.08 mm (mean 5.85 mm) post-op (C) (4–6mo)	− 1.5–2.0 mm (mean -1.62 mm) (T) − 0.3–0.6 mm (mean − 0.5 mm) (C) (1y)	< 3 mm	T: 4 wound dehiscences (17.4%) C: 2 bad split (11.8%), 1 wound dehiscence (5.9%) Wound dehiscence managed with antimicrobial rinse and systemic antibiotics Bad split managed with titanium mini screw and operation resumed. No severe infection, neurosensory disturbance, bleeding
Naenni et al. 2017 [21], Basler et al. 2018 [22]	RCT	27	51.85 ± 29.7	6 light smokers (≤ 10 cig/day) (22.2%) 21 non-smokers (77.8%)	3 treated periodontal disease (11.1%) 24 periodontally healthy (88.9%)	pDBBBM + rCM	pDBBM + non-resorbable membrane	16(T) 11(C)	Simultaneous	100%	NR	6mo	3.46 ± 0.52 (T) 2.82 ± 0.50 C)	− 2.23 ± 1.21 mm (T) − 0.14 ± 0.79 mm (C) (6mo)	NR	T: 4 wound dehiscences resolved within 4w C: 1 dehiscence resolved within 3mo, 1 dehiscence persisted till 6mo at re-entry Treated with local disinfecting agents once a week for 4w and later once a mo
Wessing et al. 2017 [23]	RCT	49	38.6 ± 15.3 (T) 48.9 ± 17.0 (C)	11 light smokers (≤ 10 cig/day) (22.4%) 38 non-smokers (77.6%)	6 treated periodontal disease (12.2%) 43 periodontally healthy (87.8%)	pABG + pDBMM + rCM	pABG + pDBMM + non-cross-linked rCM	24 (T) 25 (C)	Simultaneous	100%	NR	6mo	NR	NR	NR	33 pts: swelling (67.3%) 19 pts: redness (38.8%) 11 pts: wound dehiscence (22.4%) 6 pts membrane exposure (12.2%) No reported infection
Arunjaroensuk et al. 2018 [24]	RCT	48	51.22 ± 16.19	NR	NR	pABG covered with biphasic calcium phosphate bone + resorbable PLA membrane	pABG covered with biphasic calcium phosphate bone + rCM	30 (T) 30 (C)	Simultaneous	100%(T) 100%(C)	NR	6 mo	3.22 ± 1.00 mm (T) 3.42 ± 0.85 mm (C)	− 34.3 ± 23.85% (T) − 34.8 ± 23.68%(C) (6mo)	NR	Minor gingival inflammation and membrane exposure noted, all sites recovered uneventfully
Meloni et al. 2018 [25]	Prospective CS	45	52.1 (24–78)	13 moderate smokers (28.8%)	FMPI < 25% and FMBoP < 25%	pABG + pDBBM + rCM + Ti pins	NR	63	Simultaneous	100%	NR	3y	NR	NR	4–6 mm	6 pts: CM exposure after 1–2w Treated with 0.5% CHX gel twice a day for 3w, complete soft tissue healing
Benic et al. 2019 [26]	RCT	24	62.0 (43.5–78.6) (T) 58.1 (28.7–78.8) (C)	None with heavy smoking (> 20cig/day)	No active periodontal disease	DBBM block + rCM + fixation pins	pDBBM + native bilayer CM + fixation pins	12 (T) 12 (C)	Simultaneous	100%	NR	6mo	3.38 ± 0.59 mm (T) 2.73 ± 0.69 mm (C)	− 0.68 ± 0.82 mm/− 22.5 ± 30.9% (T) − 2.21 ± 0.98 mm/− 81.8 ± 27.4%(C) (6mo)	NR	T: 1 pt mucosal dehiscence, 1 pt swollen mucosa C: 1 pt mucosal dehiscence, 1 pt swollen mucosa
Meloni et al. 2017 [27], 2019 [28]	Prospective CS	18	56.8 (24–78)	8 light smokers (≤ 10 cig/day) (44.4%) 10 non-smokers (55.6%)	No active periodontal disease FMPI < 25% and FMBoP < 25%	pABG + pDBBM + rCM + Ti pins	NA	55	Staged	100%	NR	1y, 3y	5.03 ± 2.15 mm (7mo)	NR	≤ 4 mm (mean 3.07 ± 0.54 mm)	3 pts: CM exposure after 2w Treated with 0.5% CHX gel twice a day for 3w, complete soft tissue healing
Mendoza-Azpur et al. 2019 [29]	RCT	42	49.62 ± 10.22 (38–67) (T) 55.06 ± 10.78 (36–69)	None smoking more than 10cig/day	No untreated periodontal disease	Ramus block ABG + pDBBM + rCM	pDBBM + rCM	31 (T) 34 (C)	Staged	100%	NR	18–21mo	5.1 ± 0.87 mm (T) 5.6 ± 1.35 mm (C) (6mo)	NR	< 4 mm	T: 15 pts (68.1%) swelling 15 pts (68.1%) haematoma 7 pts (31.8%) minor sensory disturbance 1 pt (4.5%) infection 4 pts (18.1%) graft exposure C: 10 pts (50%) swelling 4 pts (20%) haematoma 1 pt (5%) minor sensory disturbance 2 pts (10%) graft exposure Sensory disturbances treated with vitamin B, resolved within 6mo Graft exposure occurred 3–4mo postop, treated with 0.5% CHX gel, 0.12% CHX rinse, 1 g augmentin bd 7d
Solakoglu et al. 2019 [30]	RCT	20	59.6 ± 10.5 (38.8–78.3)	All non-smokers	11 treated periodontal disease (55%) 9 periodontally healthy (45%)	Particulate allograft (Maxgraft) + PRGF + rCM	Particulate allograft (Puros) + PRGF + rCM	NR	Simultaneous	NR	NR	5mo (4–7mo)	NR	NR	NR	No postoperative complications, except for 3 pts who experienced slight wound dehiscences and slightly delayed wound healing
G F Tresguerres et al. 2019 [31]	RCT	28	67.1 (T) 64.5 (C)	Smokers and non-smokers 31	NR	Corticocancellous freeze dried bone allograft (iliac block) + PRGF membrane	Cancellous freeze dried bone allograft (iliac block) + PRGF membrane	53 (T) 39 (C)	Staged	100%	NR	2y	NR	− 19.27 ± 2.31 mm (T) − 29.17 ± 2.58 mm (C) (4mo)	< 4 mm	2 partial graft exposures None with significant pain, no infection Dehiscences sealed with PRGF membrane
Bartols et al. 2018 [32]	RCT	30	43.1 ± 17.2 (T) 51.5 ± 17.3 (C)	NR	NR	pABG + pDBBM + rCM	Ramus shell ABG + pABG	10 (T) 14 (C)	Staged	80% (T) 100% (C)	NR	1y	2.20 ± 0.86 mm (preop) to 9.00 ± 1.20 mm (postop) (T) 2.67 ± 0.61 mm (preop) to 8.93 ± 1.05 mm (postop) (C)	− 0.68 ± 0.98 mm (T) − 0.94 ± 0.99 mm (C) (1y)	≤ 3 mm	T: 5 of 15 sites loss of bone graft (1 due to infection, 4 due to complete resorption) 5 sites wound dehiscence 1 site inflammation (pus) at graft 1 donor site hypaesthesia C: 1 of 15 sites loss of bone graft due to infection 0 sites wound dehiscence 1 site inflammation (pus) at graft 0 donor sites hypaesthesia
Deeb et al. 2016 [33]	Retrospective cohort	52	NR	None smoking more than 20cig/day	NR	Particulate allograft + pDBBM (tunnel incision)	Particulate allograft + pDBBM + non-resorbable PTFE membrane (crestal incision)	18 (T) 22 (C)	Staged	NR	NR	6mo	NR	NR	NR	T: 4 of 21 sites wound dehiscence/membrane exposure 4 sites graft loss C: 16 of 31 sites wound dehiscence/membrane exposure 12 sites graft loss
Ersanli et al. 2016 [34]	Prospective cohort	32	NR	NR	NR	Ramus block ABG + pDBBM + rCM	Symphyseal block ABG + pDBBM + rCM	28 (T) 17 (C)	Staged	94.11% (T) 96.42% (C)	94.11% (T) 96.42% (C)	1y	4.36 ± 1.01 mm (T) 6.29 ± 0.86 mm (C)	− 0.80 ± 0.56 mm (T) − 0.60 ± 0.78 mm (C) (1y)	NR	T: 4 of 14 patients bleeding 4 hematoma 2 flap dehiscence 1 infection 0 numbness (donor site) C: 5 of 18 patients bleeding 5 hematoma 2 flap dehiscence 2 infection 1 numbness (persisting at the end of 1y follow-up) Bleeding treated with firm gauze compress Amoxicillin and clavulanic acid 1 g bd 10d for other complications All flap dehiscences resolved except for 1 case, treated with free gingival graft at 5th week postop
Jensen et al. 2016 [35]	Retrospective CS	171	NR	None smoking more than 10cig/day	No active periodontal disease	pABG + pDBBM + rCM (simultaneous) pABG/block ABG + pDBBM + rCM (staged)	NA	275	171pts simultaneous; 23pts staged	98.4% (simultaneous); 0% (staged)	NR	NR	NR	NR	NR	Simultaneous GBR: 2 of 240 sites early dehiscence (≤ 21d postop) 2 late dehiscence (> 21d postop) 1 early infection 4 late infection 0 sensory disturbances Staged GBR (pABG): 0 early/late dehiscence, early/late infection, 0 sensory disturbances Staged GBR (block ABG): 2 of 15 sites early dehiscence 5 late dehiscence 2 early infection 1 late infection 1 transient sensory disturbance
Lee et al. 2015 [36]	RCT	30	53.3 (31–75)	None smoking more than 20cig/day	None with advanced or untreated periodontal disease	pDBBM + non-cross-linked rCM	pDBBM + cross-linked rCM	30	Simultaneous	93.3% (T) 100% (C)	NR	16w	2.6 ± 0.8 mm (T) 2.3 ± 0.8 mm (C) 1 mm below implant platform	NR	NR	T: 2 of 30 sites wound dehiscence/membrane exposure C: 1 of 30 sites local infection 3 of 30 sites wound dehiscence/membrane exposure
Merli et al. 2018 [37]	RCT	50	56.0 ± 13.0 (T) 53.4 ± 12.4 (C)	None smoking more than 20cig/day	NR	pDBBM + rCM	Particulate alloplast + rCM	61	Simultaneous	100%	NR	3y	NR	NR	NR	T: 1 of 32 sites mucosal dehiscence 90 days postop 1 purulent exudate 60d postop 1 tingling sensation and hyposensitivity 7d postop C: 1 of 29 sites mucosal dehiscence 30d postop 2 purulent exudate 14d postop
Moslemi et al. 2016 [38]	Prospective CS	17	31–65	All non-smokers	NR	Particulate allograft + rCM	NA	17	Simultaneous	100%	100%	6mo	NR	NR	NR	0 wound dehiscence 0 infection 0 swelling spreading beyond surgical zone 9 of 17 sites extraoral swelling

*ABG* autogenous bone graft, *C* control group, *CHX* chlorhexidine, *cig* cigarette, *CM* collagen membrane, *CS* case series, *d* day, *DBBM* deproteinised bovine bone mineral, *ePTFE* expanded polytetrafluoroethylene, *FMBoP* full mouth bleeding on probing, *FMPI* full mouth plaque index, *IV* intravenous, *mo* month, *NA* not applicable, *NR* not reported, *No* number, *pABG* particulate autogenous bone graft, *pDBBM* particulate deproteinised bovine bone mineral, *PRGF* plasma rich in growth factors, *PLA* polylactic acid, *pt(s)* patient(s), *rCM* resorbable collagen membrane, *RCT* randomised controlled trial, *T* test group, *Ti* titanium, *w* week, *y* year

### Surgical procedure and biomaterials used

A total of 11 studies used the staged GBR approach only [16, 18–20, 27–29, 31–34], two studies included both staged and simultaneous GBR [17, 35], and the remaining ten studies performed GBR with simultaneous implant placement [21–26, 30, 36–38]. Onlay grafts were used in eight studies, of which six studies obtained grafts from intra-oral donor sites (ramus/symphysis/zygoma) [16, 18–20, 29, 34], one used extra-oral donor sites (iliac crest) [31], while the remaining study used a xenograft (deproteinised bovine bone mineral (DBBM)) block [26]. The most commonly used particulate bone substitute material in the included studies was DBBM. A majority of the studies also used resorbable collagen barrier membranes [16–30, 32, 34–38], three used a non-resorbable expanded polytetrafluoroethylene (ePTFE) membrane [21, 22, 33], one used a resorbable polylactic (PLA) membrane [24], and one used a plasma rich in growth factors (PRGF) membrane [31].

### Post-surgical complications

**Fig. 2:**
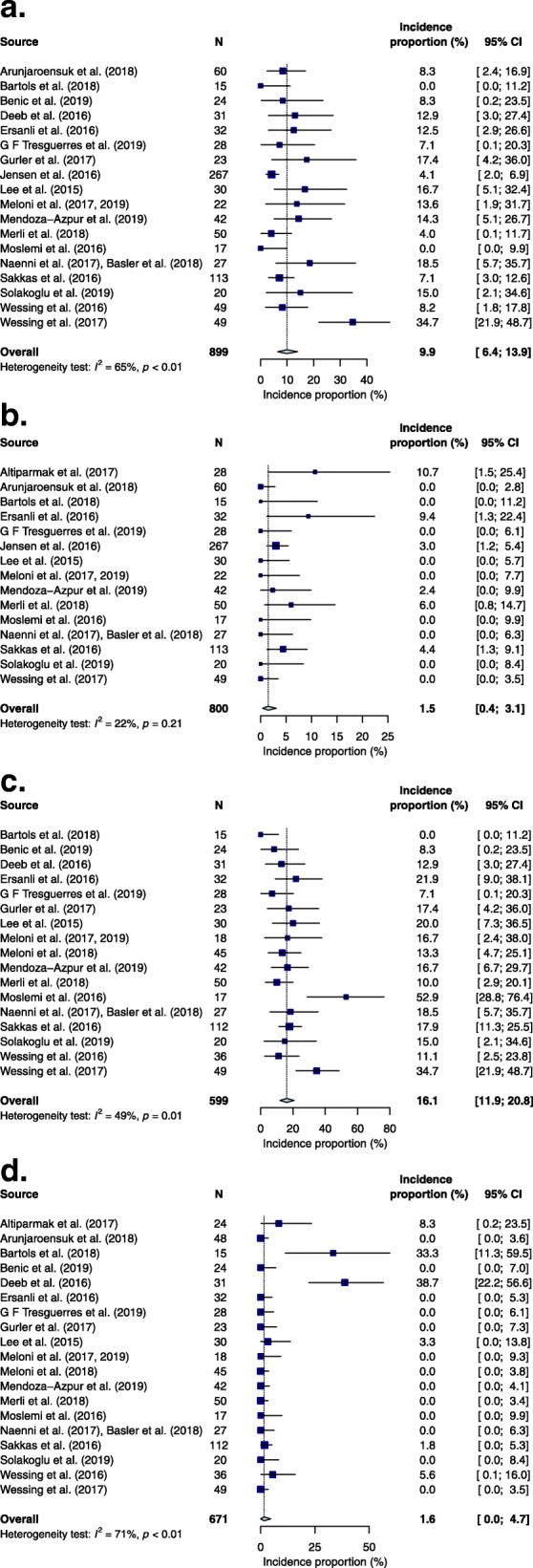
**a** Forest plot presenting weighted mean incidence proportion of minor wound dehiscences per augmented site. **b** Forest plot presenting weighted mean incidence proportion of minor infections per augmented site. **c** Forest plot presenting weighted mean incidence proportion of total minor complications at the augmented site per patient. **d** Forest plot presenting weighted mean incidence proportion of total major complications at the augmented site per patient. **e** Forest plot presenting weighted mean incidence proportion of neurosensory alterations at donor site per patient

Minor wound dehiscence was defined as sites where the surgical incision reopened, resulting in a slight gaping wound or where the barrier membrane was exposed but resolved with wound care. The site-level weighted mean incidence proportion of such minor wound dehiscence occurring at augmented sites was 9.9% [95% CI = 6.4, 13.9, *P* < 0.01] (Fig. 2a). Minor infections consisted of sites with localised suppuration or swelling that resolved with antiseptics and/or antibiotics, and this occurred at a weighted mean incidence proportion of 1.5% [95% CI = 0.4, 3.1, *P* = 0.21] at a site-level (Fig. 2b). Both wound dehiscence and minor infections could be cumulatively classified as minor complications and the overall patient-level weight mean incidence proportion was 16.1% [95% CI = 11.9, 20.8, *P* = 0.01] (Fig. 2c).

Major complications referred to persistent or worsening infections, or graft failure which resulted in the need for its partial or total removal the site of bone augmentation. The overall weighted mean incidence proportion of these major complications was 1.6% [95% CI = 0.0, 4.7, *P* < 0.01] at a patient level (Fig. 2d). Neurosensory alterations at the donor site were also reported in studies utilising autologous grafts and included symptoms such as lip paraesthesia and negative pulp sensitivity to the lower incisors. Such alterations had a weighted mean incidence proportion of 7.0% [95% CI = 1.3, 15.5, *P* < 0.01] at a patient-level (Fig. 2e) and could reportedly last for more than 6 months [34]. Funnel plots reported no publication bias except on reporting major complications (Additional file 2).

Subgroup analysis was done based on the type of study, type of bone graft used (particulate or block), type of membrane used (resorbable or non-resorbable), and type of GBR technique (staged or simultaneous) (Additional file 3). Site-level analysis showed that the use of block grafts resulted in a significantly higher incidence proportion of minor infections at augmented sites (5.7% [95% CI 1.8, 11.1, *P* = 0.12]) compared to particulate grafts (0.4% [95% CI 0.0, 1.5, *P* = 0.80]). On the other hand, the type of membrane used did not seem to have any effect on the incidence proportion of any post-surgical complication. A staged GBR procedure also resulted in a statistically significant higher site-level incidence of minor infections (4.2% [95% CI 1.6, 7.5, *P* = 0.22]) compared to simultaneous GBR (0.6% [95% CI 0.0, 1.8, *P* = 0.52]). This was also mirrored at the patient-level analysis where the incidence proportion of major complications for staged GBR stood at 5.2% [95% CI 0.1, 17.6, *P* < 0.01]) ,compared to simultaneous GBR (0.0% [95% CI 0.0, 0.8, *P* = 0.98]).

### Management of post-surgical complications

The management of post-surgical complications was only reported in thirteen studies [16, 17, 19–22, 24, 25, 27–29, 31, 34]. When a minor wound dehiscence occurred, the included studies reported common management regimes that consisted of either the use of antiseptics, systemic antibiotics, or site repair with autogenous tissue or growth factors (Table 1). Three studies advised patients to institute home care management by applying 0.5% chlorhexidine gel twice a day for 3 weeks at the dehisced site [25, 27, 28]. Two studies nursed wound dehiscence sites by recalling patients once a week for 4 weeks for professional local disinfectant application, and once a month thereafter until complete resolution [21, 22]. Two studies reported management of wound dehiscences with exposed graft materials by removing the sequestered bone [16] and treating the site with a combination of an antimicrobial mouth rinse and systemic antibiotics [16, 20]. Two studies reported uneventful and spontaneous healing of dehisced sites [17, 24]. The need for additional surgical intervention to manage minor wound dehiscences has also been looked at. While one study treated all minor wound dehiscences immediately with a PRGF membrane [31], another study treated all wound dehiscences with an initial course of systemic amoxicillin and clavulanic acid for 10 days and only proceeded to graft a dehisced site with a free gingival graft at the fifth week after surgery due to non-resolution [34]. One study reported late complications emerging only 3 to 4 months after the initial GBR, and they were treated with 0.5% chlorhexidine topical gel application, 0.12% chlorhexidine rinse, and a 1 week course of amoxicillin and clavulanic acid [29]. Minor infections such as suppuration were treated either only with systemic antibiotics [34] or with the addition of a chlorhexidine mouth rinse [16]. Regardless of which treatment strategy being employed, all these management regimes resulted in complete soft tissue healing, except in one study cohort where one case of minor dehiscence persisted until re-entry surgery 6 months later [21, 22], and in two studies where there were isolated instances of total graft exposure which necessitated complete removal of the graft and membrane [16, 17]. When graft failure occurred, the two reported cases in one study declined further bone augmentation procedures [16], and the one case in the second study experienced another surgical failure after a repeated augmentation procedure, with success reported only after the third time when autogenous bone was used instead of a combination of autogenous and demineralised bovine bone mineral [17]. For patients who reported having neurosensory alterations at the donor sites, they were given regular follow-up review appointments [16, 19, 29], with one study prescribing patients with a course with vitamin B [29]. These studies reported complete neurosensory resolution between 2 and 6 months.

### Heterogeneity test

There was low heterogeneity among studies reporting the incidence proportion of minor infections at a site-level (*I*^2^ = 22%, *P* = 0.21). There was moderate heterogeneity (*I*^2^ = 49%, *P* = 0.01) among studies reporting on the total minor complications at a patient-level, moderately high heterogeneity (*I*^2^ = 65%, *P* < 0.01) among studies reporting on minor wound dehiscence at a site level, and high heterogeneity (*I*^2^ = 71%, *P* < 0.01; *I*^2^ = 75%, *P* < 0.01) among studies reporting on the total major complications and neurosensory alterations at a patient-level respectively.

### Risk of bias assessment

**Fig. 3:**
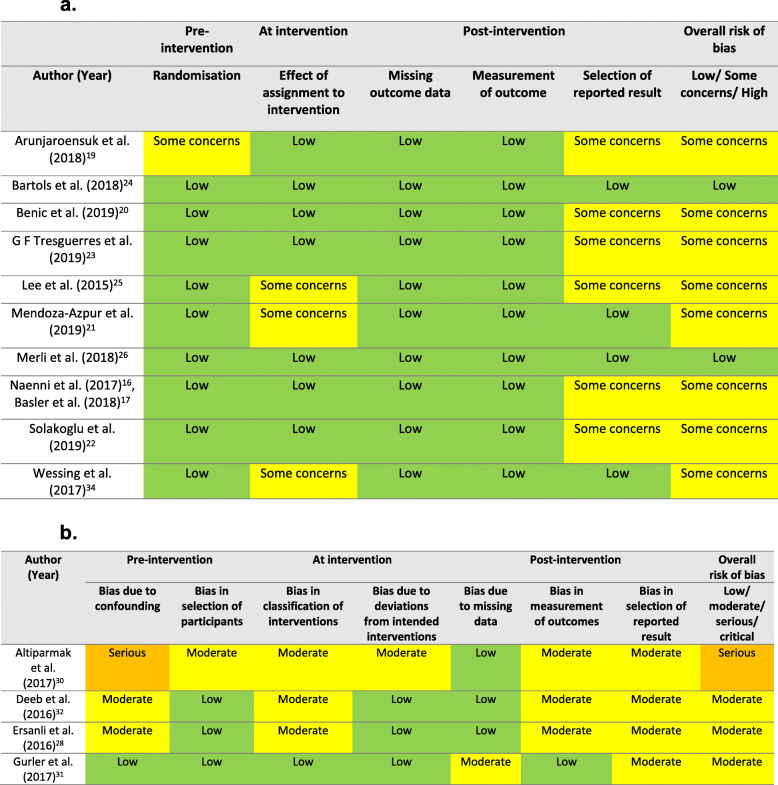
**a** Risk of bias of included randomised controlled clinical trials. **b** Risk of bias of included observational studies

Ten randomised controlled clinical trials were assessed using the ROB 2 (Fig. 3a). Two observational studies [32, 37] had an overall low risk of bias, and eight observational studies [21–24, 26, 29–31, 36] had some concerns in bias. Four observational studies were assessed using the ROBINS-I tool (Fig. 3b). Three studies [20, 33, 34] had an overall moderate risk of bias, while one study had a serious risk of bias [18].

### Histomorphometric analysis in sites with post-surgical complications

Out of the five studies reviewed, three studies involved human subjects [44–46], while two were animal studies [47, 48] (Table 2). For these studies, histological assessment was conducted at sites experiencing wound dehiscence and graft exposures post-GBR. Graft failure and its subsequent membrane and graft loss was observed in one study. The majority of the studies involved the use of a particulate bone graft [44–47], except for one which used an autogenous onlay graft [48]. The membranes of choice were either a resorbable collagen membrane or an ePTFE membrane. None of the studies included morphometric analysis of sites with post-GBR complications.

**Table 2:** Features of included studies

Author/year	Study model	Defect	Healing period	Device	Clinical/gross presentation of dehiscence	Histological presentation of dehiscence
Friedmann et al. 2001 [44]	16 human subjects	Chronic	7mo	pDBBM covered with bovine type I collagen cross-linked CM	Within first 14d: Exposure of CM 7 out of 10 dehiscences completely closed within following 14 days 1 site completely covered by new gingiva with some exfoliated pDBBM after 4w 2 sites completely closed only 14d after the 6w review None of the dehisced sites showed any signs of inflammation, degradation, swelling, redness, exudation at any time point	At 7mo: No difference in appearance between membranes with and without exposure Possible that bacteria adhered to exposed membrane surfaces, but no granulocytic infiltration observed at 7mo Tissue apposition and ingrowth into gaps between the collagen layers occurred independent of membrane exposure
Friedmann et al. 2002 [45]	14 human subjects	Chronic	7mo	pDBBM covered either with bovine type I collagen cross-linked CM or ePTFE membrane	With bovine type I CM: Within first 14d: 9 out of 14 sites had exposure of CM, complete closure after another 4w None of the dehisced sites showed any signs of inflammation, degradation, swelling, redness, exudation at any time point Uneventful delayed pattern of gingival healing With ePTFE membrane: Exposure during and after initial healing period Some required premature retrieval of membrane due to non-healing One site required complete removal of graft and membrane due to infection and severe inflammation	At 7mo: Dehiscence occurrence had no resulting difference in bone quality
Friedmann et al. 2015 [46]	12 human subjects	Chronic	6mo	Biphasic calcium phosphate covered either with non-cross-linked CM or cross-linked CM	7 out of 13 sites had compromised healing (wound dehiscence and/or graft exposure)	At 6mo: Low remodelling rates Osteogenesis detected in 4 out of the 7 sites, with the remaining displaying missing or minimal osteogenesis In cases with absent osteogenesis, the graft material was covered predominantly by dense collagen tissue populated by multinuclear cells resembling either osteoclasts or epitheloid giant cells with intermingled mononuclear cells
Bornstein et al. 2007 [47]	6 beagle dogs	Saddle-type defect created	8, 16w	pDBBM + pABG (1:1 ratio) covered with either porcine type I and II CM or cross-linked porcine type I and III CM	At 1w: 2 out of 6 sites had exposure of CM and signs of local inflammation At 6w: Wound closure complete, no inflammation observed	At 8w: Less bone regeneration, remnants of cross-linked collagen barrier with signs of inflammation Dome shape contour of newly formed bone not apparent
Donos et al. 2002 [48]	30 Wistar rats	None (graft placed directly on ridge)	15, 30, 60, 120, 180d	ABG covered with either resorbable membrane or ePTFE	At 15d: With ePTFE membrane: Membrane exposure in 5 out of 6 sites With resorbable membrane: Membrane exposure in 4 out of 6 sites At 30d: With ePTFE membrane: Membrane lost in 1 site, membrane exposure in remaining 4 out of 5 sites With resorbable membrane: Membrane exposure in 3 out of 6 sites At 60d: With ePTFE membrane: Microimplant lost in 5 out of 6 sites making histological preparation impossible. Membrane exposure in remaining site With resorbable membrane: Membrane exposure in 4 out of 6 sites At 120d: With ePTFE membrane: Microimplant, graft, membrane lost in 3 sites. Only membrane was lost in remaining 3 sites With resorbable membrane: Microimplant and graft lost in 2 sites. Membrane exposure in 3 out of the remaining 4 sites At 180d: With ePTFE membrane: Microimplant, graft, membrane lost in 5 sites. Only membrane was lost in remaining site With resorbable membrane: Microimplant, graft, membrane lost in 2 sites	At 15d: With ePTFE membrane: Graft contained osteocytes and had maintained its height. Periphery of graft had scalloped appearance due to resorption lacunae. Zone of connective tissue with numerous leucocytes, lymphocytes, plasma cells between graft and membrane. Richly vascularised granulation tissue between graft and recipient site With resorbable membrane: Exposed parts of membrane were fragmented and surrounded by connective tissue that had inflammatory infiltrate. Non-exposed parts had preserved their structure and embedded in connective tissue At 30d: With ePTFE membrane: Graft contained osteocytes, had scalloped surface. Inflammatory infiltrate between membrane and graft. Layer of fibrous connective tissue between the recipient site and graft (as opposed to newly formed immature trabecular bone in continuity between recipient site and graft, at the site with no dehiscence) With resorbable membrane: Membrane broken into large fragments, encapsulated by fibrous connective tissue with inflammatory cells. Bone graft had significant resorption especially at lateral edges of graft. Remaining parts surrounded by connective tissue with inflammatory infiltrate, and there was no bone continuity between recipient site and graft. Resorption of recipient bone had occurred (as opposed to newly formed mature trabecular bone in continuity between recipient site and graft, at the site with no dehiscence) At 60d: With ePTFE membrane: Bone graft had disappeared. Resorption of recipient bone had occurred With resorbable membrane: Small fragments of membrane could be detected. Bone grafts had empty lacunae. Two sites had graft that maintained its height, while two others the lateral border of the graft was exposed and inflammatory infiltrate was observed adjacent to the graft. Bone continuity between graft and recipient site was not observed in any site with membrane exposure At 120d: With ePTFE membrane: Bone graft partially or almost completely resorbed but two specimens had bone continuity between remaining parts of graft and recipient bone With resorbable membrane: Small fragments of membrane present adjacent to non-exposed lateral borders of graft. Exposed portion of graft had empty osteocyte lacunae, and height and width of graft was reduced due to resorption. Recipient bone also exhibited resorption. No bone continuity between graft and recipient site (as opposed to bone contact, at sites with no dehiscence) At 180d: With ePTFE membrane: Only 1 site had bone graft remaining, which contained osteocytes and had maintained its height. Bone continuity between graft and recipient bone was observed With resorbable membrane: Bone graft completely resorbed in 1 site. In remaining 3 sites, operated area covered by oral mucosa with very small remnants of membrane

*ABG* autogenous bone graft, *CM* collagen membrane, *d* day, *ePTFE* expanded polytetrafluoroethylene, *m* month, *pABG* particulate autogenous bone graft, *pDBBM* particulate deproteinised bovine bone mineral, *w* week

Of the three human studies, biopsies for histological analysis were obtained during second-stage surgeries, which took place between 6 and 7 months after the initial surgeries. When a resorbable collagen membrane was used [44–46], most sites with clinical evidence and history of wound dehiscences had complete wound closure within 6 weeks from the initial surgery. In spite of the exposure, none of the sites presented with evidence of inflammation, degradation, swelling, erythema, or suppuration throughout the period of study. Conversely, when a non-resorbable ePTFE membrane was used [45], some dehiscences failed to heal even after the initial healing period and certain sites required premature removal of the membrane and bone graft.

Two studies showed no difference in terms of bone quality between the membranes with or without membrane exposure at 6 to 7 months [44, 45]. In addition, tissue apposition and ingrowth into the gaps between the collagen layers had occurred independent of membrane exposure. Though there was a possibility of bacterial contamination of the membrane during the period of wound dehiscence, no granulocytic infiltration was noted at 7 months. However, there was one study that observed that sites with membrane exposure had lower remodelling rates, with some sites displaying missing or minimal osteogenesis [46]. Sites with a lack of osteogenesis showed the graft material being covered by dense collagen tissue populated with multinucleated cells.

Two animal studies were included in this review. In the first study, wound dehiscence and inflammation occurred in two out of six sites within the first week post-surgery, and complete wound closure was achieved by the end of 6 weeks. Histological analysis at eighth week revealed that sites with history of wound dehiscence during the initial phase of healing displayed less bone regeneration [47]. The other study compared the use of a resorbable membrane against an ePTFE membrane in GBR and analysed the sites histologically at 15, 30, 60, 120, and 180 days. At 15 days, the periphery of the bone graft exhibited a scalloped appearance due to resorption lacunae, and a zone of connective tissue with inflammatory infiltrate and richly vascularised granulation tissue was observed at the interface between the recipient site and the bone graft. At 30 days, sites with membrane exposure had a layer of fibrous connective tissue between the recipient bone and the graft, instead of newly formed trabecular bone in contact with each other as observed at sites that were complication-free. Furthermore, if a resorbable membrane was exposed, it was observed that the membrane had disintegrated into large fragments which were then separately encapsulated by fibrous connective tissue in the presence of inflammatory cells. The associated bone graft materials also had more significant resorption especially at the lateral edges. On top of that, resorption of the recipient native bone site was also observed. The progressive resorption of the remnant bone grafts continued from day 60 to 120. At 60, 120, and 180 days, none of the membrane exposed sites except one displayed any bone continuity between the graft and recipient native bone [48].

## Discussion

### Primary findings

Minor wound dehiscence, with a weighted mean incidence proportion of 9.9% [95% CI 6.4, 13.9, *P* < 0.01] at site-level, is the most common post-surgical complication that occurs after GBR. It can lead to early or late membrane exposure, contamination, infection, and partial or total loss of the graft. Membrane exposure leads to a significant detrimental negative effect on horizontal bone gain, with sites without membrane exposure showing 74% more bone gain than those with membrane exposure [49]. Despite the relatively high incidence of wound dehiscence, post-surgical infection of the graft is relatively uncommon with a weighted mean incidence proportion of 1.5% [95% CI 0.4, 3.1, *P* = 0.21] for minor infections per augmented site and 1.6% [95% CI 0.0, 4.7, *P* < 0.01] for major complications at augmented site per patient. Nonetheless, any complication will potentially lead to increased treatment time and cost to the patient and thus should be avoided or managed in a timely manner should it occur.

The use of cross-linked and non-resorbable membranes has reportedly been associated with a greater incidence of wound dehiscence because of its tendency to revert back to its original shape, instead of staying adapted to the graft site [50, 51]. A recent systematic review reported an overall membrane exposure rate of 22.7% in simultaneous GBR procedures with no significant difference between resorbable and non-resorbable membranes [9]. This is also confirmed by the findings in this systematic review which showed no difference in any post-surgical complication regardless of membrane type.

The use of block grafts and staging a GBR procedure was shown to result in a higher prevalence proportion of post-surgical complications. This may be explained by the fact that sites which require block grafts or staged GBR procedures usually require a larger volume of horizontal augmentation, which can potentially result in greater flap tension, dehiscence, and subsequent post-surgical complications. The use of a block graft, especially those derived only from cortical bone, may reduce the chances of its revascularisation and vitality, which can confer a higher risk of graft necrosis and infection [52].

### Secondary findings

Guided bone regeneration is a predictable surgical procedure when there is proper adaptation and stabilisation of the membrane and maintained flap closure during the healing phase. The animal studies in this review showed that wound dehiscence resulted in less bone regeneration, graft resorption, and a lack of bone continuity between the recipient site and the grafted bone [47, 48]. Resorption of the recipient bone can occur as well in such defects. A mild inflammatory reaction, probably a foreign body reaction, was observed 15 days post-surgically. However, when the membrane was exposed to the oral environment, a layer of fibrous connective tissue between the recipient site and the bone graft was almost always observed. The exposure of an ePTFE and a resorbable collagen membrane was constantly associated with a dense chronic inflammatory infiltrate, and the exposed resorbable collagen membrane had almost completely resorbed by 30 days. Persistent unresolved contamination of the membrane eventually led to high rates of graft and membrane loss. The high failure rates depicted in the animal studies are partially due to the inability to institute frequent repeated sessions of general anaesthesia to conduct post-surgical wound management. Perhaps with the frequent reviews and decontamination of the exposed site as mentioned in the earlier section, the incidence of such failures would be much reduced.

One clinical study [46] which investigated the use of non-cross-linked and cross-linked collagen membranes reported lower bone remodelling rates and compromised osteogenesis in sites with dehiscence, which were consistent with the findings from the animal studies. However, the other two clinical trials in this review [44, 45] seemed to suggest that the composition and quality of new bone formation was independent of any episodes of wound dehiscence. This could have been due to the type I collagen cross-linked membranes used in those studies, which might be more resistant to attack by mammalian and bacterial collagenase. Secondly, in spite of the dehiscences, the membranes appeared clinically intact during the healing period, and all the dehisced sites were completely healed after institution of a strict antiseptic regime where patients were instructed to use chlorhexidine gel three times a day at exposed areas and reviewed weekly. None of the dehisced sites in these two studies had any clinical sign of inflammation, swelling, erythema, or suppuration.

### Clinical implications

Many published studies have demonstrated that width of keratinised mucosa, flap thickness, flap tension, vestibular depth, type and size of alveolar defect, and the materials used [53] were related to the occurrence of wound dehiscence. Out of the various factors that could influence success of the GBR procedure, several authors have highlighted the upmost importance of a tension-free flap closure in ensuring complete wound closure and an uneventful healing phase [54–56] and one study in particular emphasised that this factor is more critical than flap thickness in determining surgical success [57]. For most GBR sites, vertical releasing incisions are employed to allow for flap advancement but at sites with large volume of added bone grafts, such incisions alone might still not be adequate to provide for a tension free flap closure. This can be overcome by the addition of periosteal releasing incisions which can allow for an additional flap extension of 5.5 mm compared to vertical releasing incisions alone [58]. Other authors have also suggested modifying the recipient site flap management by creating longer horizontal extensions at the apical extents of the flap, having secondary flap reflection after membrane fixation, and/or employing a double flap incision design which involves suturing the periosteum before suturing the flap in order to achieve flap passivity [59–61]. During the pre-operative preparative phase, operators should have a clear grasp of the anatomy of the planned surgical site. This knowledge would come in handy especially while designing an access flap for sites planned for large volume augmentation. Operators can then consider and explore the use of more intricate flap designs such as mobilising lingual flap for the mandible or incorporating a sub-orbicularis muscle flap manipulation at the anterior maxilla region to better facilitate in tension free flap closure [56, 62, 63]. However, these advanced surgical techniques are not complication-free and patients might run into risks of developing neurosensory alterations. Though generally uncommon, one should not overlook the possibility of mental and lingual nerve paraesthesia occurring as a result of poor flap preparation and management. A dome-shaped incision around the mental nerve foramen has been suggested to avoid mental nerve damage, while vertical incisions should not be performed on the lingual aspect for fear of lingual nerve damage [61]. Most of these neurosensory alterations are transient and resolve within 8 weeks [64, 65] and can be monitored with mechanosensory measures such as pin prick tests, gentle touch, and two-point discrimination thresholds using linear analogue scales or with patient-reported difficulty speaking and/or eating [66]. A lack of improvement after 3 months indicates that normal function may not be achieved [67], and a referral to an appropriate specialist is recommended for further management, which ranges from conservative to surgical reconstruction. In the unfortunate event that nerve transection is suspected during the surgery, it will require immediate nerve exploration and repair by an experienced surgeon. Besides modifying flap designs, periodontal plastic surgeries such as soft tissue grafts, frenectomies, and vestibuloplasties can be considered to prevent wound dehiscence by achieving a variety of goals such as increasing the flap thickness or keratinised mucosal width, or to reduce the muscle pull from a shallow vestibule or aberrant frenum, but there is limited evidence that these techniques prevent wound dehiscence [68].

Wound dehiscence can be classified based the size of the exposure and whether there is presence of exudate, namely small exposures equal or less than 3 mm without purulent exudate, larger exposures without purulent exudate, and membrane exposure with purulent exudate [69]. According to the included studies in this review, when wound dehiscence occurs, any purulent exudate if present has to be drained and loose mobile graft biomaterial should be removed to facilitate wound closure and healing. The site should subsequently be thoroughly irrigated with chlorhexidine solution. This should be followed up with either twice daily application of chlorhexidine gels [25, 28, 41] or rinses [41] to reduce plaque accumulation at the wound sites. Weekly reviews [41] should be arranged to nurse the surgical site with the use of cotton tips or swabs soaked with chlorhexidine until the overlying epithelium barrier is formed. While two studies reported the use of adjunctive systemic antibiotics at the time of early exposure [16, 20], one must bear in mind the need for judicious prescription of antibiotics and should only be reserved for cases with abscesses formation, purulent exudation, and/or systemic involvement such as fever and malaise. Abscess formation is indicative of possible bacterial contamination and may also occur in the early stages after GBR. Membrane removal, graft curettage, and antibiotic cover should be carried out in such cases [69]. For larger exposures (more than 3 mm), the risk of secondary infection may be higher as secondary wound healing will take a longer time, and such defects may require surgical intervention to remove the membrane and flap advancement to re-suture the dehisced flaps together [69]. Late exposures occurring after 3 months from the time of the surgery may also be indicative of an underlying infection. In such cases, antibiotic therapy with close reviews can be considered first [29] before considering the removal of the membrane and any infected graft material [70]. A common antibiotic regimen would be amoxicillin/clavulanic acid (875/125 mg) twice a day for 7 days [29].

In spite of these measures, varying success rates are expected—some cases achieved spontaneous wound coverage [21, 23, 25, 28, 39, 41], while for others, dehiscence persisted up to 6 months post-surgery, resulting in the need for additional bone augmentation at the re-entry stage [21, 22]. A re-entry stage at 6 months has been suggested, irrespective of wound dehiscence occurring, unless total graft failure occurs prior to that. A reassessment of the surgical site is then made on whether there is any need for further bone augmentation.

### Agreement and disagreement with previous studies

A recently published systematic review reported an overall soft tissue complication rate of 16.5% [10], which is similar to the overall incidence proportion of minor complications as reported in this study. However, that review found that the use of a resorbable membrane had a higher weighted complication rate when compared to the use of non-resorbable membranes, contrary to the findings from this study which found no difference in post-surgical complications irrespective of membrane material. Our results also concurred with that of another systematic review which also found similar complication rates between non-resorbable (13.9%) and resorbable (13.6%) membranes [9]. The difference in post-surgical complications for non-resorbable membranes between these studies may be attributed to other confounders such as the timing of removal of the membrane, as its delayed removal may have resulted in an increased incidence of post-surgical complications. Furthermore, the types of soft tissue complications and severity of wound dehiscence that occurred in their qualitative analyses, which were not reviewed in the other two systematic reviews, could also play a role in influencing the differences in results. A review also investigated the incidence of donor site complications and noted sensory alterations of the mucosa occurring 18.57% and 8.19% of the time when the ramus and symphysis were used as donor sites respectively [71]. The grafts harvested from these donor sites were used as block grafts rather than particulate grafts, which required creating osteotomies with a surgical bur or trephine. While that review did not conduct a meta-analysis, the complication incidence was notably higher from the results of this present review. This could be because older studies from more than 20 years ago were included in that review, and the osteotomy designs might have been more aggressive or extensive. In addition, 3-dimensional imaging (which would be otherwise be mandatory today) might not be available or being used by surgeons then to identify and avoid proximal vital structures, thus leading to higher incidences of neurosensory alterations.

### Limitations and recommendations for future research

In the present review, some caution is advised in interpreting the main findings due to moderate to high heterogeneity among studies when reporting post-surgical complications. The type of study also gave rise to moderate to high heterogeneity when reporting on some of the post-surgical complications (Additional file 3). In addition, post-surgical complications are often not reported in a standardised manner, which gave rise to a reporting bias as shown in the risk of bias analysis. For example, only two studies reported post-surgical complications such as extra-oral swelling [17, 38], two studies reported post-surgical bleeding [20, 34], and one study reported redness as a complication without much elaboration [23]. The negative impact of tobacco smoking on wound healing has been well documented in the literature [72]. However, smoking as a factor could not be isolated for subgroup analysis as most studies did not compare post-surgical complications between smokers and non-smokers. Only one study analysed smoking as a contributory factor and showed that tobacco smoking increased the incidence of post-surgical complications [16]. There was a distinct lack of reporting post-surgical complications at the donor site, and most of the included studies in this review reported only on neurosensory alterations. Furthermore, soft tissue complications such as wound dehiscence and infections at the donor site were often not reported separately and thus precluded a subgroup analysis. This study also quantitatively assessed the complication incidence of neurosensory alterations at donor sites which were used for block grafts only. With the advent of minimally invasive techniques such as shaving or scraping these donor sites to obtain particulate autogenous bone, it would be interesting to evaluate if the post-surgical complications would be markedly reduced when compared to conventional techniques such as autogenous bone harvesting using a surgical bur or trephine.

The heterogeneity in the study designs, the different follow-up periods, and the grafting materials used were the main confounding factors in this review. The ePTFE membranes were the only non-resorbable membranes analysed in this review, but this membrane, especially the titanium-reinforced type, is no longer commercially available. Hence, other non-resorbable membranes such as dense-PTFE membranes should be compared to resorbable membranes in future reviews. There is also a need for studies to report post-surgical complications in a systematic manner. Factors such as the dimensions of the initial and post-surgical ridge width, different grafting materials, assessing complications according to different donor sites, and classifying complications according to early or late complications should be examined in future clinical trials in order to better understand the occurrence of post-surgical complications.

## Conclusion

The increased demand for implants in resorbed or narrow ridges has given rise to various surgical techniques to augment ridges horizontally. The techniques involved in horizontal bone augmentation can potentially result in post-surgical complications such as soft tissue wound dehiscence, membrane exposure, partial or total loss of graft material, and neurosensory alterations. Horizontal GBR is a relatively safe procedure with a low incidence of major complications. Neurosensory alteration is not an uncommon post-surgical complication when autogenous bone is harvested from a donor site and hence caution is advised. Minor complications occur relatively more commonly, with minor wound dehiscence occurring at an incidence proportion of 9.9% at a site-level. When such complications occur, it requires timely intervention and follow-ups. Exposure of the barrier membrane as a result of wound dehiscence can lead to poorer wound healing and can result in a reduction in the quality and amount of bone regeneration. It is critical that the augmented surgical site remains free of contamination and that the membrane maintains its barrier function in order to allow the underlying bone graft to be populated by osteogenic cells. It is suggested that post-surgical complications should be systematically reported in future clinical trials for research purposes.

## Supplementary Information

**Additional file 1.** Summary of excluded studies.

**Additional file 2.** Funnel plots on publication bias.

**Additional file 3.** Subgroup analysis of post-surgical complications.

## Data Availability

All data are available in the manuscript.

## Abbreviations

GBR: Guided bone regeneration
CI: Confidence interval
CENTRAL: Cochrane Central Register of Controlled Trials
MeSH: Medical Subject Headings
PRISMA: Preferred Reporting Items for Systematic Reviews and Meta-Analyses
ROBINS-I: Risk of Bias in Non-randomized studies—of Interventions
ROB-2: Risk-Of-Bias tool 2.0
DBBM: Deproteinised bovine bone mineral
ePTFE: Expanded polytetrafluoroethylene
PTFE: Polytetrafluoroethylene
PRGF: Plasma rich in growth factors
