# Supplementary material for: Clinical and histological sequelae of surgical complications in horizontal guided bone regeneration: a systematic review and proposal for management
Source: Int J Implant Dent. 2020 Nov 26;6:76. doi: 10.1186/s40729-020-00274-y (PMC7688776; doi:10.1186/s40729-020-00274-y)
Supplement: Supplementary file 1 — Additional file 1. Summary of excluded studies. [file 40729_2020_274_MOESM1_ESM.docx]

**Additional file 1**

**Summary of excluded studies**

| Excluded study | Reason for exclusion |
| --- | --- |
| Al-Abedalla et al. (2015) | Did not differentiate types of bone defects |
| Dastaran et al. (2015) | Oral presentation |
| Dastaran et al. (2019) | Did not differentiate types of bone defects |
| Deeb et al. (2015) | Oral presentation |
| Deluiz et al. (2016) | Did not differentiate types of bone defects |
| Eisenbraun and Tarasenko (2015) | Oral presentation |
| Eisenbraun and Tarasenko (2017) | Oral presentation |
| Gomes et al. (2016) | Immediate implants included |
| Guiol et al. (2017) | Conference proceeding |
| Gultekin et al. (2017) | Vertical defects included |
| Jung et al. (2017) | GBR group≤15 patients |
| Kim et al. (2015) | Did not differentiate types of bone defects |
| Krasny et al. (2015) | Did not differentiate types of bone defects |
| Lee (2017) | Included cases not limited to horizontal GBR on healed ridges |
| Lizio et al. (2016) | Vertical defects included |
| Moukrioti et al. (2019) | Did not use barrier membrane |
| Nam et al. (2017) | Vertical defects included |
| Sakkas et al. (2018) | Cases not limited to horizontal GBR |
| Sumida et al. (2015) | Vertical defects included |
| Thoma et al. (2019) | Did not differentiate types of bone defects |
| Wen et al. (2018) | Did not report complications |
