# Supplementary material for: Clinical and histological sequelae of surgical complications in horizontal guided bone regeneration: a systematic review and proposal for management
Source: Int J Implant Dent. 2020 Nov 26;6:76. doi: 10.1186/s40729-020-00274-y (PMC7688776; doi:10.1186/s40729-020-00274-y)
Supplement: Supplementary file 2 — Additional file 2. Funnel plots on publication bias. [file 40729_2020_274_MOESM2_ESM.docx]

**Additional file 2**

**Figure 1. Funnel plot of meta-analysis reporting on site-level minor wound dehiscences.**


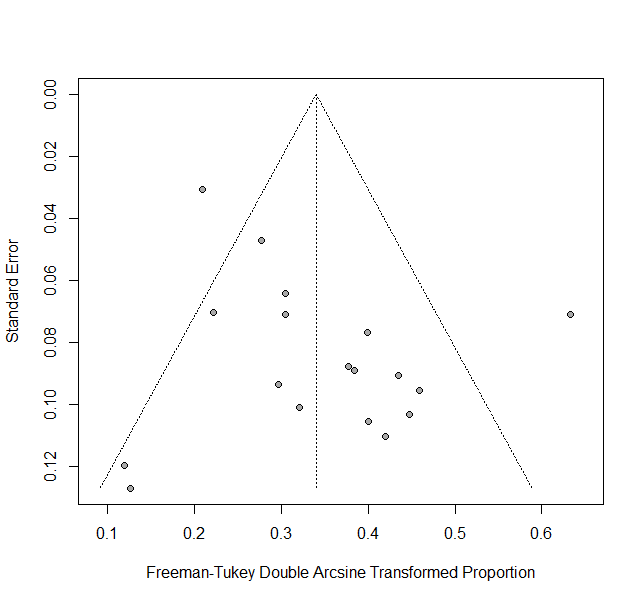


Test for funnel plot asymmetry: p-value = 0.448

**Figure 2. Funnel plot of meta-analysis reporting on site-level minor infections.**


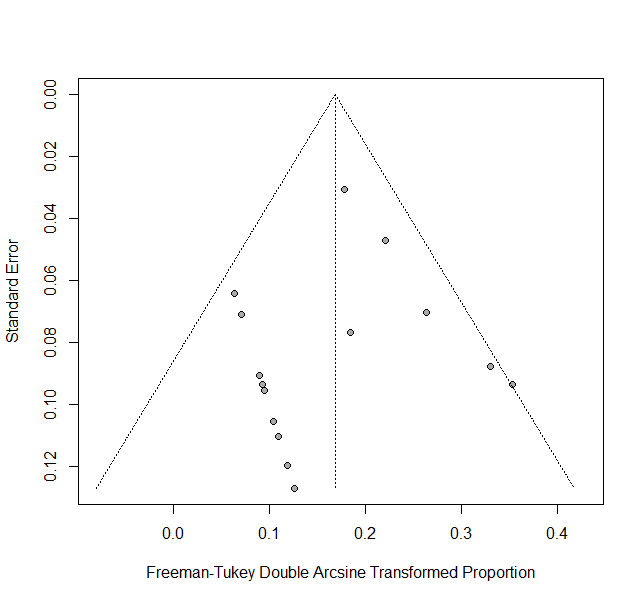


Test for funnel plot asymmetry: p-value = 0.692

**Figure 3. Funnel plot of meta-analysis reporting on patient-level minor complications.**


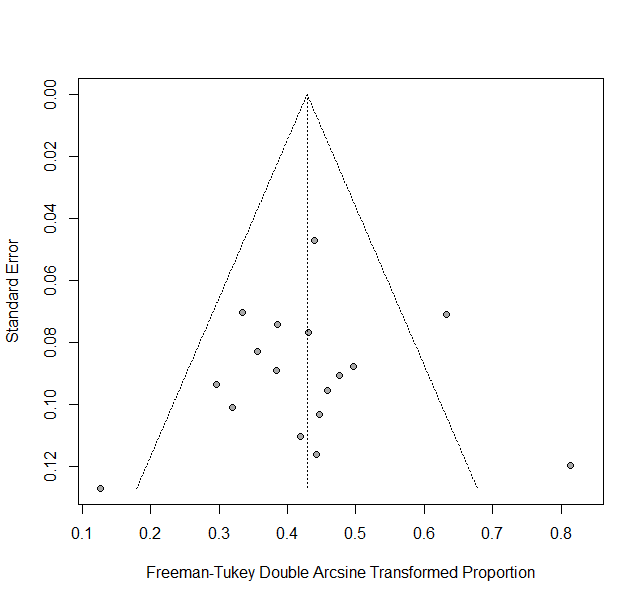


Test for funnel plot asymmetry: p-value = 0.8393

**Figure 4. Funnel plot of meta-analysis reporting on patient-level major complications.**


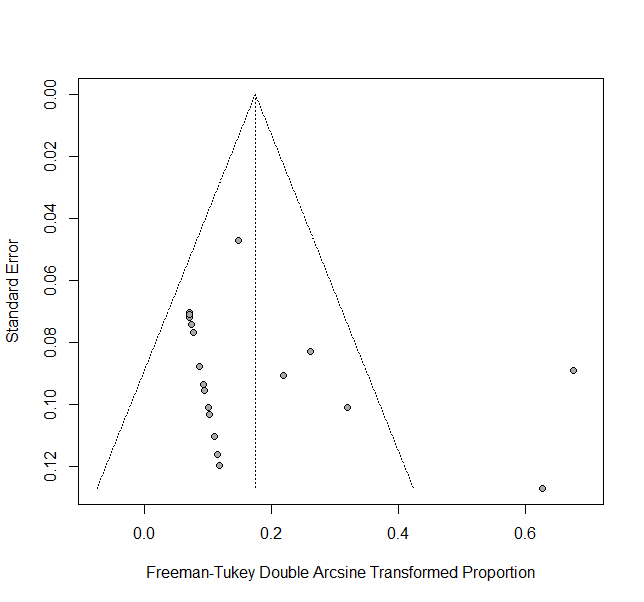


Test for funnel plot asymmetry: p-value = 0.033

**Figure 5. Funnel plot of meta-analysis reporting on patient-level neurosensory alterations.**


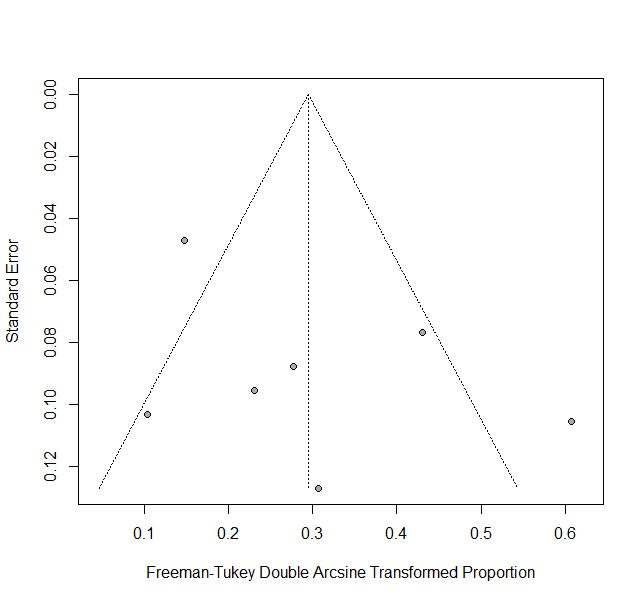


Test for funnel plot asymmetry: p-value = 0.5619
